# Development of a model to predict the age at breast cancer diagnosis in a global population

**DOI:** 10.1038/s41598-024-53108-x

**Published:** 2024-06-15

**Authors:** Ha Young Kim, Jimmy Mullaert, Ambre Tondreau, Boyoung Park, Roman Rouzier

**Affiliations:** 1 Department of Health Sciences, College of Medicine, Hanyang University, Seoul, Korea; 2 Institut Curie, PSL Research University, INSERM, U900, Saint Cloud, France; 3 Department of Medicine, College of Medicine, Hanyang University, Seoul, Korea; 4 Department of Surgery, Centre François Baclesse, Caen, France

**Keywords:** Breast cancer, Cancer epidemiology, Diagnosis, Disease prevention, Public health

## Abstract

Knowing the mean age at diagnosis of breast cancer (BC) in a country is important for setting up an efficient BC screening program. The aim of this study was to develop and validate a model to predict the mean age at diagnosis of BC at the country level. To develop the model, we used the CI5plus database from the IARC, which contains incidence data for 122 selected populations for a minimum of 15 consecutive years from 1993 to 2012 and data from the World Bank. The standard model was fitted with a generalized linear model with the age of the population, growth domestic product per capita (GDPPC) and fertility rate as fixed effects and continent as a random effect. The model was validated in registries of the Cancer Incidence in Five Continents that are not included in the CI5plus database (1st validation set: 1950–2012) and in the most recently released volume (2nd validation set: 2013–2017). The intercept of the model was 30.9 (27.8–34.1), and the regression coefficients for population age, GDPPC and fertility rate were 0.55 (95% CI: 0.53–0.58, *p* < 0.001), 0.46 (95% CI: 0.26–0.67, *p* < 0.001) and 1.62 (95% CI: 1.42–1.88, *p* < 0.001), respectively. The marginal R^2^ and conditional R^2^ were 0.22 and 0.81, respectively, suggesting that 81% percent of the variance in the mean age at diagnosis of BC was explained by the variance in population age, GDPPC and fertility rate through linear relationships. The model was highly accurate, as the correlations between the predicted age from the model and the observed mean age at diagnosis of BC were 0.64 and 0.89, respectively, and the mean relative error percentage errors were 5.2 and 3.1% for the 1st and 2nd validation sets, respectively. We developed a robust model based on population age and continent to predict the mean age at diagnosis of BC in populations. This tool could be used to implement BC screening in countries without prevention programs.

## Introduction

The epidemiology of breast cancer (BC) in different countries is determined by the economic development of the country, environmental factors and the ethnicity of the population^1,2^. Differences between incidence and mortality rates for BC are particularly common when comparing data from developed countries to those from low- and middle-income countries (LMICs)^1,3^. The upward trends in the BC burden in some countries can be attributed to the aging and growing population as well as rapid economic development^4^. Among the factors influencing cancer epidemiology, economic factors, especially economic development, play an important role^5,6^. On the international scale, the relationship between cancer disparity and economic development has already been well studied. Health care systems in LMICs may face strong incentives and pressure to adopt health care interventions such as BC screening, which are well established in high-resource settings, with implicit assumptions that the benefits demonstrated in more developed countries will also apply to less developed countries^7,8^. BC screening could have an impact on LMICs if it increases BC awareness and early detection^8^. However, many countries lack a national program because national protocols regarding the appropriate age range for screening are unavailable^5,9,10^.

Population-based cancer registries are a core component of cancer control strategies. Registries systematically collect information from multiple sources on all reportable neoplasms occurring in a geographically defined population^11,12^. Worldwide, cancer registries have been shown to be critical for determining the cancer burden, conducting research and planning and implementing cancer control measures. Incidence data collected from cancer registries in different parts of the world provide an opportunity for examining epidemiological transitions in some countries, particularly the relationship between cancer incidence and age on a wider scale^13,14^. Despite these acknowledged utilities of cancer registration, cancer registries are not yet an integral part of cancer control in most LMICs^5,6,8,9^. Even descriptive statistics are insufficient, as data coverage remains incomplete in many low-resource economies. This suggests that BC estimates in these countries may be biased and that the implementation of a generalizable model could contribute to promoting more efficient BC (correct age range) screening and informing policy appropriately. It may be hypothesized that available data from registries and socioeconomic factors could be used to predict age at BC diagnosis in LMICs and therefore be used to establish BC screening or policies.

Taking advantage of publicly available data from cancer registries and socioeconomic factor data, the aim of the present study was to develop and validate a model to predict the mean age at diagnosis of BC at the country level.

## Materials and methods

### Data sources and quality control

**Figure 1:**
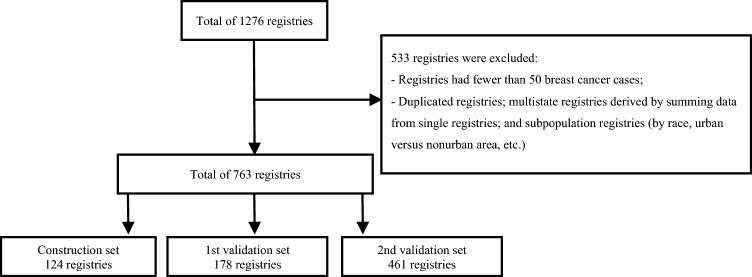
Flow chart.

We conducted a registry-based study on incident cases of female BC in different countries. We used public data extracted from the CI5plus database and CI5 Volume V to XII on BC incidence among females and Cancer Incidence in Five Continents by the International Agency for Research on Cancer (IARC, the datasets are available on https://ci5.iarc.fr/^14^. CI5 data provide information on the number of patients in different registries according to patient volume and summary, sex, age group, type of cancer, and population distribution. Through this approach, we were able to identify the number of BC patients by age group and obtain the average age of the patients according to each registry. We used the CI5plus database, which contains BC incidence data for 122 selected populations for a minimum of 15 consecutive years between 1993 and 2012, to develop a model for predicting the mean age at diagnosis of BC. Data on the growth domestic product per capita (GDPPC) and fertility rate over the study period for each country were obtained from the World Bank and the datasets used this study are available the World Bank (https://www.worldbank.org/en/home). All fertility rate data were available, while less than 5% of GDPPC data were missing (imputation was made by linear interpolation). The model was evaluated on two validation sets: the 1st validation set included 178 registries (not included in the CI5plus database) of CI5 Volume V to XI from 1993 to 2012, and the 2nd validation set included CI5 Volume XII data from 2013 to 2017 published recently (October 2023) (Fig. 1). We excluded registries that had fewer than 50 cases of BC, and to avoid data duplication, we excluded multistate registries derived by summing data from single registries and subpopulation registries (races, urban versus nonurban areas, short term, etc.). The 1st and 2nd validation sets and both the 1st and 2nd validation sets included 15, 5 and 11 new countries, respectively, which were not included in the construction set. None of the data used in the construction set were used in the validation sets.

### Statistical analysis

To develop the model, we used the CI5plus database and explored the mean age of female BC patients on different continents by year. We also evaluated the correlation between the mean age of the population and the mean age at diagnosis of BC by continent to choose the best multivariate model. For this, age is given in quinquennia of age (0–4, 5–9,…, 85 + years) by year and by population, so we calculated an approximated mean age of each class for each year of this period and each registry, as reported by Bidoli et al.^13^. We classified the results according to the Sustainable Development Goal (SDG) regions as defined by the United Nations Statistics Division and used for the Sustainable Development Goals Report.

The statistical unit was the registry to capture as much variability as possible. A linear mixed model was fitted with age of the population as a fixed effect and registries on the SDG as a random effect. The performance of the model in the construction set was evaluated using marginal and conditional R-squared (R^2^) values, which indicated the percentage of the variance in age for BC that was explained by the model. Fixed effect estimations and variance components are reported along with 95% confidence intervals. The model was then first externally validated in registries of the Cancer Incidence in Five Continents that are not included in the CI5plus database. We performed a second validation using the model with data from CI5 Volume XII (data from 2013 to 2017 released in October 2023). We predicted the mean age at diagnosis of BC according to the mean age of the population, SDG region, GDPPC and fertility rate by using the model developed using the CI5plus database. Correlations between predicted and observed values were assessed using Pearson’s correlation coefficient (R^2^).

To analyze the difference between the mean age at BC diagnosis from the prediction model and the observation, the mean absolute error, relative error (predicted mean age at BC diagnosis—observed mean age at BC diagnosis)/observed mean age at BC diagnosis, mean relative error and mean relative error percentage (MREP) were used for the different analyses. Analyses were conducted for all registries, by country and by region as defined by the United Nations. We evaluated the correlation between the relative error and the number of BC patients in each registry in the 1st validation set.

We conducted 2 additional analyses to further evaluate the modeling of the mean age at BC diagnosis. First, we evaluated the impact of the GDPPC and fertility rate on the performance of the model for Sub-Saharan Africa (validation in the 1st and 2nd validation sets combined). Second, we included a random sample of 25–75% of the most recent dataset (CI5 Volume XII (data from 2013 to 2017)) and evaluated the difference in performance between the original model and each new model (1000 iterations) on the remaining data of the most recent CI5 dataset.

R statistical software (version 3.5.2, R Foundation for Statistical Computing) was used. A *p* value < 0.05 was considered to indicate statistical significance.

### Ethical approval

This is an observational study. “Development of a Model to Predict the Age at Breast Cancer Diagnosis in a Global Polulation” Research Ethics Committee has confirmed that no ethical approval is required.

## Results

Table 1 shows the characteristics of the registries used for the construction and validation sets. The number of registries in each SDG region varied between the construction set and the validation sets, with more registries from North America and Europe in the construction set (68%), while registries from Sub-Saharan Africa accounted for 1% of the construction and 2 and 3% of the 1st validation set and 2nd validation set, respectively.

**Table 1:** Description of regions by continent compared between the construction set in the CI5 summary and the validation sets in the CI5 volumes.

	Construction set				1st Validation set				2nd Validation set			
	N	%	Median/Mean age (SD) of the population	Median/Mean age (SD) at breast cancer	N	%	Median/Mean age (SD) of the population	Median/Mean age (SD) at breast cancer	N	%	Median/Mean age (SD) of the population	Median/Mean age (SD) at breast cancer
Sub-Saharan Africa	20	1	19.8/19.9 (0.43)	46.6/46.1 (2.51)	13	2	30.0/28.5 (7.81)	49.2/52.4 (6.82)	10	2	23.8/26.1 (5.68)	51.9/51.8 (4.04)
Central and Southern Asia	30	1	28.6/28.9 (1.90)	52.4/52.3 (1.39)	54	8	30.1/31.4 (5.54)	53.2/54.4 (5.22)	26	6	30.2/30.3 (2.96)	52.7/52.4 (2.95)
Eastern and South-Eastern Asia	417	13	36.0/36.1 (5.42)	52.4/53.0 (3.64)	81	11	35.6/35.3 (4.78)	54.4/55.3 (4.95)	181	39	39.0/39.5 (3.73)	53.4/53.7 (2.64)
Europe and Northern America	2196	68	38.8/38.4 (3.94)	61.2/60.6 (2.90)	466	66	38.3/37.6 (5.18)	62.0/61.5 (4.02)	184	40	41.9/41.9 (2.82)	62.5/62.3 (1.42)
Latin America and the Caribbean	138	4	29.2/30.0 (3.79)	56.0/55.7 (2.49)	67	10	32.0/32.5 (5.96)	56.7/56.5 (4.59)	25	5	34.4/34.4 (2.36)	58.2/58.3 (1.79)
Oceania	230	7	36.0/35.7 (2.64)	60.1/59.6 (2.24)	3	< 1	32.6/31.9 (3.03)	56.2/55.9 (1.33)	17	4	38.4/37.3 (5.01)	61.4/59.9 (2.71)
Northern Africa and Western Asia	200	6	30.3/29.9 (4.03)	55.7/56.0 (3.54)	21	3	32.6/31.4 (5.55)	51.2/52.6 (5.62)	18	4	33.2/32.6 (4.25)	53.9/54.1 (2.84)

**Figure 2:**
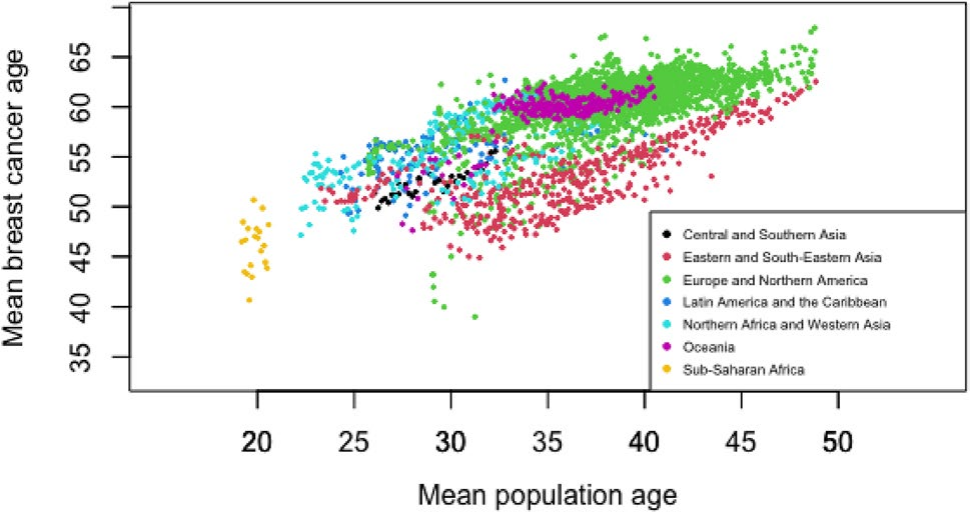
Correlation between the mean age at breast cancer diagnosis and the population mean age by continent according to the CI5 summary.

The correlation between the mean age of the population and the mean age at diagnosis of BC according to all the registries by continent according to the CI5 summary is shown in Fig. 2. Pearson’s correlation coefficient was 0.66 (95% CI: 0.65–0.68, *p* value < 2e−16). The mean age at diagnosis of BC was also strongly correlated with the GDPPC and fertility rate according to univariate analysis (Pearson’s correlation coefficient: 0.54 (95% CI = 0.52–0.57, *p* value < 2e−16) and − 0.37 (95% CI = − 0.40–0.34, *p* value < 2e−16) respectively). These findings reinforced the choice of a random effects model for SDG region and fixed effects model for population age, GDPPC and fertility rate.

From the construction set, we constructed a linear model with random effects (Table 2). Age, GDPPC and fertility rate were independently associated with the mean age at diagnosis of BC, with coefficients of 0.55 (95% CI: 0.53–0.58), *p* value < 0.001), 0.46 (95% CI: 0.26–0.67), *p* value < 0.001) and 1.62 (95% CI: 1.42–1.82), *p* value < 0.001), respectively (Table 2). This model had marginal and conditional R^2^ values of 0.216 and 0.81, respectively. The equation derived from the model was as follows: Predicted mean age at diagnosis of BC = 30.94 + 0.55 population mean age + 0.46 GDPPC + 1.62 fertility rate + (Central and Southern Asia: − 1.5, Eastern and Southeastern Asia: − 2.6, Europe and North America: 3.5, Latin America and the Caribbean: 2.3, Oceania: 3.8, Sub-Saharan Africa: − 7.6).

**Table 2:** A generalized linear model with age of the population as a fixed effect and registries on the continent as a random effect.

	Mean breast cancer age		
Predictors	Estimates	CI	*p*
(Intercept)	30.94	27.80–34.08	** < 0.001**
Mean age of the population	0.55	0.53–0.58	** < 0.001**
GDPPC	0.46	0.26–0.67	** < 0.001**
Fertility rate	1.62	1.42–1.82	** < 0.001**
Random Effects			
σ^2^	4.72		
τ_00 SDG_	14.79		
ICC	0.76		
N_SDG_	7		
Observations	3231		
Marginal R^2^/Conditional R^2^	0.216/0.810		

*GDPPC* growth domestic product per capita, *SDG* Sustainable Development Goal, *ICC* intraclass correlation coefficient.

Significant values are in [bold].

**Figure 3:**
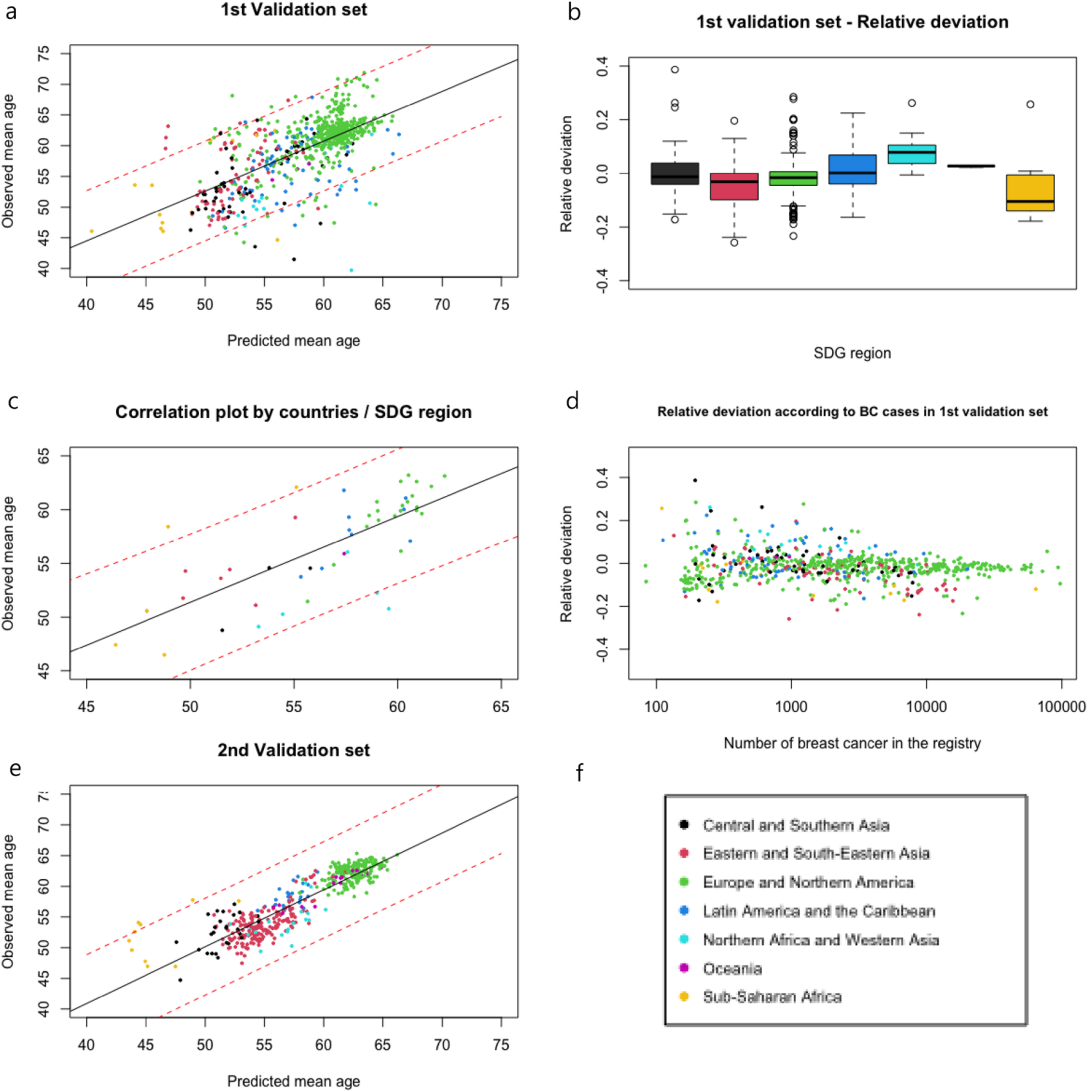
The correlation between the predicted and observed mean age at breast cancer diagnosis. (**a**) Correlation plot for the 1st validation set, R^2^ = 0.64 (*p* < 0.001); (**b**) boxplot of relative deviation by Sustainable Development Goal (SDG) region; (**c**) correlation plot by country from each continent, R^2^ = 0.75 (*p* < 0.001); (**d**) relative deviation according to BC cases in the 1st validation set (log scale); (**e**) correlation plot for the 2nd validation set, R^2^ = 0.89 (*p* < 0.001). The black line and dashed red lines represent the reference line indicating perfect calibration and the 95% prediction interval, respectively. (**f**) SDG region legend.

We validated this model in independent datasets. The correlation between the predicted and observed mean age at diagnosis of BC in the 1st validation set is shown in Fig. 3a (3f.: showed SDG region legend of Fig. 3). The correlation was good: R^2^ = 0.64 (*p* < 0.001). The MREP was 5.2% (standard deviation: 6.1). The distribution of relative errors was similar across continents (Fig. 3b). Correlations between the predicted and observed mean ages at diagnosis of BC by country are presented in Fig. 3c. The correlation was high (R^2^ = 0.75; *p* < 0.001), and the MREP was 4.1% (corresponding to a mean absolute error of 2.3 years), suggesting the exportability of the model at the country level for determining BC screening age. Interestingly, greater errors were observed for South Africa and Libya, the two countries with the highest human development level in Africa. The relative error according to the number of BC cases is represented in Fig. 3d. As the size of the registry increased, the relative deviation converged around the true underlying effect size.

For the 2nd dataset, which consisted of data for the years 2013–2017, the correlation between the predicted and observed mean age at diagnosis of BC is shown in Fig. 3e. The correlation was high (R^2^ = 0.89; *p* < 0.001). The MREP was 3.1% (standard deviation: 2.7), suggesting the exportability of the model for recent years.

The data of 31 countries not included in the construction set were tested separately. The correlation between the predicted and observed mean ages at BC diagnosis was high (R^2^ = 0.83; *p* < 0.001). The MREP was 6.4% (standard deviation: 9.9) for these countries (7.5% and 4.9% for new countries in the 1st and 2nd validation sets, respectively), suggesting the exportability of the model for new countries.

We conducted an additional analysis to further evaluate the modeling of the mean age at BC diagnosis in Sub-Saharan African countries. Including the GDPPC and fertility rate in the model greatly improved model performance. The correlation and mean relative error ranged from R^2^ = 0.52 and an MREP of 10.3% (standard deviation: 7.9) without GDPPC or fertility rate in the model to R^2^ = 0.7 and an MREP of 6.8% (standard deviation: 4.9) with GDPPC and the fertility rate in the combined validation set suggesting that these simple socioeconomic factors should be added to the model. We conducted a second additional analysis to evaluate whether adding a random sample of 25–75% of the most recent dataset (CI5 Volume XII (data from 2013 to 2017)) to the model improved performance and evaluated the difference between the original model and the new model in the remaining registries of the CI5 Volume XII. In the 1000 iterations, the predictions were highly correlated (R^2^ = 1), the absolute mean difference between the predictions was 0.18 years, and the maximal difference was between 1.7 and 2.56 years (Supplemental Fig. 1).

## Discussion

We developed an independent and original model to predict the mean age at diagnosis of BC in this study. The association between the mean age at BC diagnosis and geographic region was similar to that reported in previous studies^15–17^. Therefore, the model to predict the mean age at BC diagnosis in this study, which has a high explanatory coefficient reflecting the characteristics of a country, can aid in the development and implementation of BC prevention programs in all countries, especially LMICs. Specifically, we demonstrated in our study that incorporating the GDPPC and fertility rate in the model improves its pertinence for Sub-Saharan countries. Mammography screening programs typically target women aged 50–74 years^14^ according to IARC screening test protocol reports. Cancer registries operating in low- and middle-income settings may face particular challenges following international registration standards^12,18^. For example, a lack of coverage by pathology laboratories or difficulty accessing diagnosis records reduces the percentage of microscopically verified cases^19–21^ and results in postponement of the incidence date as determined according to the ENCR recommendations, which define the incidence date as the date of first histological or cytological confirmation of malignancy^18,22,23^. Addressing the escalating burden of BC in LMICs demands a multidimensional approach that encompasses prevention, early detection and improved care for affected patients. Although early detection through clinical breast examination (CBE) screening may not necessarily lead to a difference in mortality^24^, as demonstrated in randomized trials^25,26^, LMICs have implemented screening programs with CBE as the primary test, despite limited evidence of its effectiveness in reducing mortality. To alter the course of BC in LMICs, it is essential to combine these screening efforts with educational activities and ensure access to well-organized and affordable cancer health services. Studies identifying cost-effective and feasible service delivery strategies are pivotal in areas where primary care infrastructure and resources are highly limited. Given what is known about diagnostic delays, late-stage presentation, and the epidemiology of BC in sub-Saharan Africa, it is clear that, according to our findings, BC control policies must focus first and foremost on establishing accessible treatment for early-stage disease in younger women compared to other regions.

Bidoli previously highlighted the statistically significant relationship between population age and median age at BC diagnosis (of note, the median age and mean age at BC diagnosis are very similar; Table 1). They calculated a 42% R^2^ and claimed that a one-year increase in the median age of the population increased the median age of onset of BC by half a year^13^. We demonstrated in our study that a multivariate approach is an important method for predicting the mean age at diagnosis of BC. Random effects models are useful tools for both exploratory analyses and prediction problems and are particularly pertinent in our study because incidence rate trends of BC differ between continents^27,28^. In addition, the mean age at BC diagnosis shows a similar trend^16^. Among SDG regions, a wide variety of factors, such as race, nutrition, reproductive factors and genetic factors, should be considered^29–31^. These well-identified risk factors may be included in further studies.^30,32^ If national production levels, such as the GDPPC, are one of the important factors affecting BC incidence, they also usually impact BC screening^5,18,25,33^. This study could contribute to reducing the cancer burden and mortality of BC in many LMICs. Additionally, this study could serve as the foundation for establishing baseline data for future target age ranges in implemented screening programs across all countries. This should be performed in accordance with the mean age of the population and the predicted mean age at BC diagnosis. Figure 3a shows real differences in the age at BC diagnosis in the different SDG regions, supporting the importance of individualized BC screening for age of onset. One could argue that this difference in the age at BC diagnosis is actually caused by differences in screening age at disease onset across continents, but the magnitude of the difference in the mean age at BC diagnosis differs according to screening age at disease onset. The robust mean age at BC diagnosis prediction model can be used for BC prevention, considering intercontinental differences, and can provide a sufficient basis for the development and implementation of screening test protocols.

However, our study has several limitations that must be acknowledged. First, the most recent data were from 2017. The publication of the CI5 report determined the duration of this study, creating a gap of approximately six years from the present. Second, this study was conducted with a population approach rather than an individualized approach, without considering all known risk factors for BC. Third, there are people of various races on continents; the characteristics of each race should be considered, and the research model needs to be continuously expanded. Fourth, in this study, indices such as education level, environmental factors and factors other than the fertility rate (specific factors related to childbirth, breast density, etc.) that can represent the country were not considered. We tried to collect several indices for this study, but in many LMICs, many metrics were not collected over the study period. Future studies are warranted, but our results strongly suggest that the mean population age, GDPPC, fertility rate and SDG region encompass a large part of a country’s information, as demonstrated by the high percentage of the variance in the mean age at diagnosis of BC that is explained by the variance in our model. Finally, there are a small number of registries, a lack of control for population-based data registrations and a lack of data continuity in Africa. Data from African countries cannot be verified through several reliable public data sites, and the survey are conducted on an NGO or private medical basis, which is highly restricted in securing data. Through this study, we were able to confirm the importance of a population-based cancer registry for the different continents and compare BC incidence and incidence according to age and continental characteristics.

The strengths of this study are as follows. First, we used CI5 volume and summary datasets from the IARC, which are reliable data collected from the population-based cancer registry of each region. This validated use of data increased the reliability of the values analyzed and calculated in this study and produced reliable results. For the statistical approach, statistical verification through the construction set and validation set confirmed significant results, and the statistical approach was appropriate. From this, we identified a simple but unprecedented measure of mean age at diagnosis of BC using a prediction formula, which was simple and applicable to all continents in the present study. In addition, if applied to countries with insufficient or low data collection capacity, appropriate screening targets and protocols in LMICs can be identified. BC screening tests need to be introduced at many LMICs, and at the same time, a system that can collect population-based data should be introduced. A large number of LMICs are located on the African continent, where BC screening programs are often carried out by NGOs or small clinics, but we do not even know if they are being carried out properly. Therefore, we believe that in countries where BC registries are not in place and BC screening programs are not being carried out properly, predicting the age range of BC could contribute greatly to preventing BC or reducing BC mortality through early detection.

## Conclusion

We proposed a validated model based on population age, SDG region, GDPPC and the fertility rate of a country to predict the average age at BC diagnosis in populations. In the future, the development of new expanded models will also be feasible for countries that can collect information on various common environmental factors based on the model presented in this study. By implementing BC screening and policies at the optimal population age in countries without prevention programs, especially in LMICs, and facilitating the development of protocols for population-based data collection related to BC, this tool can ultimately serve as a foundation to help prevent BC and reduce mortality.

### Supplementary Information

Supplementary Information.
