# Supplementary material for: Development of a model to predict the age at breast cancer diagnosis in a global population
Source: Sci Rep. 2024 Jun 15;14:13845. doi: 10.1038/s41598-024-53108-x (PMC11180172; doi:10.1038/s41598-024-53108-x)
Supplement: Supplementary file 1 — Supplementary Information. [file 41598_2024_53108_MOESM1_ESM.docx]

**Supplemental fig 1.** a) Correlation coefficients and b) absolute mean (o) and maximal (•) differences between predictions of original model and a model including 25-75% of recent data in the remaining registries of recent data (CI5 Volume XII) – 1000 iterations.

a

**
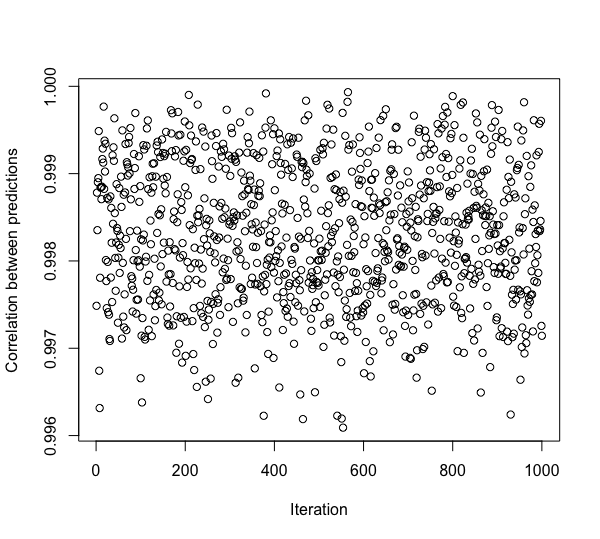
**

b

**
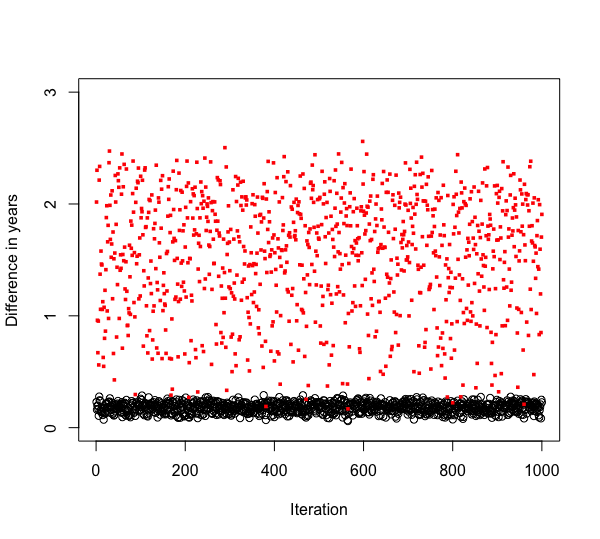
**
